# Response to: Are there two disjunct episignatures for KMT2B-related disease?

**DOI:** 10.1093/braincomms/fcae384

**Published:** 2024-12-09

**Authors:** Edoardo Monfrini, Andrea Ciolfi, Marco Ferilli, Marco Tartaglia, Alessio Di Fonzo

**Affiliations:** Dino Ferrari Center, Neuroscience Section, Department of Pathophysiology and Transplantation, University of Milan, Milan 20122, Italy; Neurology Unit, Foundation IRCCS Ca’ Granda Ospedale Maggiore Policlinico, Milan 20122, Italy; Molecular Genetics and Functional Genomics, Bambino Gesù Children’s Hospital, IRCCS, Rome 00146, Italy; Molecular Genetics and Functional Genomics, Bambino Gesù Children’s Hospital, IRCCS, Rome 00146, Italy; Molecular Genetics and Functional Genomics, Bambino Gesù Children’s Hospital, IRCCS, Rome 00146, Italy; Neurology Unit, Foundation IRCCS Ca’ Granda Ospedale Maggiore Policlinico, Milan 20122, Italy

We thank Prof. Konrad Oexle for his letter and interest in our work.^1^ His concerns are related to the ‘proclaimed’ occurrence of different epitypes (i.e. allelic episignatures) associated with different sets of variants affecting *KMT2B*. A simple reading of our paper shows that the colleague has clearly misinterpreted our considerations.^2^

In order to ‘avoid unnecessary confusion’, we address each of the raised main concerns below.

First, we made explicit that the c.1918T>A (p.S640T), c.2909G>A (p.R970Q) and c.7016G>A (p.R2339Q) substitutions were predicted as ‘benign’ or as having conflicting predictions by various *in silico* tools.^2^ Though these tools are generally highly valuable in the assessment of the functional impact of genomic variants, it should be noted that they might misinterpret variant functional relevance and clinical significance in various circumstances, particularly when considering adult-onset diseases with reduced penetrance and variable expressivity. As an example, this is the case of a class of Parkinson’s disease risk *GBA1* missense variants (e.g. E326K and T369M) that are improperly predicted as ‘benign’ by most of these tools.

Regarding Oexle’s^1^ consideration related to the occurrence of a ‘disjunct episignature’ linked with *KMT2B* variants associated with/contributing to adult-onset dystonia, as explicitly stated in our article, the tested samples carrying the four *KMT2B* variants were not classified as fitting DYT28 based on the originally identified ‘conventional’ episignature.^3^ Then, we used DNA methylation profiling to explore a differential impact shared by these four variants. Notably, the identified set of differentially methylated probes common to the eight affected individuals was not ‘proclaimed’ as defining a new ‘disjunct episignature’. Instead, our findings highlight a common differential behaviour (in terms of genome-wide DNA methylation) of the eight samples sharing rare missense *KMT2B* variants.

We thank Oexle^1^ for suggesting the adoption of analytical approaches using more stringent genome-wide significance thresholds. We note that false discovery rate (FDR) correction of *P*-values represents the most commonly used strategy in controlling errors arising from multiple testing. A long-standing wide consensus about the effectiveness of FDR in managing this specific issue stands in the scientific community.^4^ Moreover, the selection of the approach was constrained by the small sample size.

Finally, as stated in the original article, the causal association between dysregulated KMT2B function and adult-onset dystonia requires further confirmatory studies using independent pedigrees and cohorts. While the possibility that the shared DNA methylation pattern might represent a ‘contingent effect independent of KMT2B’ cannot ruled out *a priori*, by providing the identified differentially methylated probes we encouraged researchers to independently explore this association. An independent validation of our findings would represent only a first step towards the identification of a new disease-specific DNA methylation signature, which, as discussed in the article, necessarily requires a collaborative effort to consider sufficiently large/informative patient cohorts having an accurate clinical and molecular characterization.

## Data Availability

Data sharing is not applicable to this article as no new data were created or analysed.

## References

[1] Oexle K . Are there two disjunct episignatures for KMT2B-related disease? Brain Commun. 2024;6. 10.1093/braincomms/fcae383 PMC11631077 39659969

[2] Monfrini E, Ciolfi A, Cavallieri F, et al Adult-onset KMT2B-related dystonia. Brain Commun. 2022;4(6):fcac276. 36483457 10.1093/braincomms/fcac276 PMC9724767

[3] Ciolfi A, Foroutan A, Capuano A, et al Childhood-onset dystonia-causing KMT2B variants result in a distinctive genomic hypermethylation profile. Clin Epigenetics. 2021;13(1):157. 34380541 10.1186/s13148-021-01145-y PMC8359374

[4] Benjamini Y . Discovering the false discovery rate. J R Stat Soc Ser B Stat Methodol. 2010;72(4):405–416.

